# Supplementary material for: Choosing where to give birth: Factors influencing migrant women’s decision making in two regions of Thailand
Source: PLoS One. 2020 Apr 2;15(4):e0230407. doi: 10.1371/journal.pone.0230407 (PMC7117675; doi:10.1371/journal.pone.0230407)
Supplement: Attachment — Submitted filename: PLOSone.docx [file pone.0230407.s001.docx]

Dear Sir,

Thanks for invited me to review.

This is a good article that present the related factors for the pregnant women who need to make a decision to give birth in Thailand. Even for Thai people when we need to get any medication, we also need to think for the place to go too due to the health system.

The author also defined what the group of subject is and the definition for this study. In the detail in this article were arranged in each step and easy to understand and follow for the methods.

Congratulations

Prakaykaew
